# Plasma indole-3-propionic acid is a gut-derived metabolite and is associated with type 2 diabetes and cardiometabolic outcomes: Evidence from a human antibiotic intervention

**DOI:** 10.1080/19490976.2026.2736321

**Published:** 2026-09-26

**Authors:** Florine H. M. Westerbeke, Moritz V. Warmbrunn, Melany Rios-Morales, Xinmin S. Li, Melissa Y. Tian, Madelyn A. Novak, Austin M. Williams, Stanley L. Hazen, Ilias Attaye, Max Nieuwdorp

**Affiliations:** a Department of Internal and Vascular Medicine, Amsterdam University Medical Center, Amsterdam, the Netherlands; b Department of Experimental Vascular Medicine, Amsterdam University Medical Center, Amsterdam, the Netherlands; c Department of Heart, Blood, and Kidney Research, Cleveland Clinic, Cleveland, OH, USA; d Center for Microbiome & Human Health, Cleveland Clinic, Cleveland, OH, USA; e Department of Cardiovascular Medicine, Heart, Vascular, and Thoracic Institute, Cleveland Clinic, Cleveland, OH, USA; f Department of Internal Medicine, Division of Nephrology and Transplantation, Erasmus University Medical Center, Rotterdam, the Netherlands

**Keywords:** Indole-3-propionic acid, IPA, metabolite, gut microbiota, type 2 diabetes, antibiotic

## Abstract

The gut microbiota influences host metabolism through diverse metabolites, many of which have been linked to glucose homeostasis and type 2 diabetes (T2D). Understanding microbial contributions to metabolite biosynthesis is essential for developing dietary and microbiota-targeted T2D prevention and treatment strategies. We performed targeted plasma metabolomics in individuals with T2D and healthy controls, all receiving histidine supplementation, before and after gut microbiota suppression using 7-day broad-spectrum antibiotic treatment. Associations between pre-antibiotic metabolite levels and fecal metagenomics-derived gut microbiota composition were examined using co-abundance network analysis and Random Forest modeling. Indole-3-propionic acid (IPA) was the only gut-derived metabolite differing between groups before antibiotics, with lower levels in T2D and higher levels associated with reduced T2D odds. Antibiotic treatment reduced IPA to near-undetectable levels in both groups, confirming its predominantly microbial origin. Beyond established inverse associations with BMI and glycemic markers, we found a novel inverse correlation between IPA and glycemic variability, consistent with a protective association with T2D. Plasma IPA was associated with gut microbiota beta diversity. IPA-associated species clustered within a single co-abundance module, but did not include known IPA producers, suggesting plasma IPA is influenced by broader microbial community composition rather than IPA-producing capacity of individual taxa alone. This study provides direct human evidence that plasma IPA is virtually exclusively gut microbiota-derived in individuals with T2D, extending prior findings in healthy populations. It highlights IPA’s relevance to metabolic health and T2D, and guides future research on dietary and microbiota-targeted strategies to modulate IPA, advancing T2D prevention and treatment.

## Graphical abstract

**Figure:**
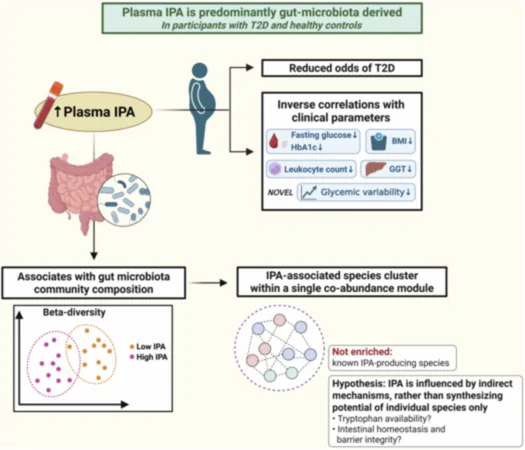

## Introduction

The rising prevalence and incidence of diabetes over the past decades presents a major global health concern. Between 1990 and 2021, the global prevalence nearly doubled from 3.2% to 6.1%, of which 96.0% is attributable to type 2 diabetes (T2D).^1^ Concerningly, diabetes prevalence is projected to continue increasing in the next few decades, reaching an estimated 9.8% by 2050, affecting more than 1.3 billion individuals worldwide.^1^ Given that T2D accounts for the vast majority of diabetes cases, these trends underscore the urgent need to better understand the underlying mechanisms of T2D and to develop effective preventive and therapeutic strategies.

The gut microbiota is increasingly recognized as a contributor to the development and progression of cardiometabolic diseases, including T2D.^2^ Individuals with T2D show a distinct gut microbial composition compared with healthy controls, suggesting a role in disease development and progression, largely through the production of diet-derived metabolites.^3^ Well-established examples include short-chain fatty acids,^4-6^ branched-chain amino acids,^7-9^ bile acids,^10^ ^,^ ^11^ and trimethylamine *N*-oxide (TMAO),^12-17^ all associated with glucose homeostasis, insulin sensitivity, and T2D risk. In addition, aromatic amino acid-derived metabolites have received growing attention, and many of these metabolites have been independently associated with cardiometabolic disease.^18^ ^,^ ^19^ Tryptophan is an essential aromatic amino acid,^20^ metabolized by both the host and gut microbiota, through three primary pathways, producing indole derivatives, kynurenines, and serotonin.^18^ ^,^ ^21^ The indole pathway is largely mediated by gut microbial metabolism, producing derivatives linked to improved intestinal barrier function, immune regulation, and glucose metabolism.^22-24^ The kynurenine and serotonin pathways are primarily host-mediated, although both can be influenced by the gut microbiota.^25-27^ Phenylalanine and tyrosine, two other aromatic amino acids, can be metabolized by gut microbiota into various metabolites including phenylacetic acid, phenylacetylglutamine (PAGln), and *p*-cresyl sulfate (PCS), which have similarly been linked to insulin resistance, T2D and atherothrombotic disease.^28-32^ These metabolites exert their effects not only by modulating insulin signaling and glucose metabolism, but also through systemic and intestinal immune modulation, influencing intestinal barrier integrity and oxidative stress.^23^ ^,^ ^33-35^ Together, this illustrates the diverse mechanisms through which gut microbially produced metabolites may contribute to cardiometabolic health and T2D.

Despite growing evidence linking the gut microbiota to T2D, current knowledge is primarily derived from preclinical and observational studies, limiting the ability to disentangle the directionality of host-microbiota interactions and direct translation to humans. Understanding the contribution of gut microbiota to plasma metabolite levels in T2D subjects and their impact on host health is essential for the development of targeted preventive and therapeutic strategies. Antibiotic-induced suppression of the gut microbiota provides a useful approach to address this, as broad-spectrum antibiotics transiently eliminate most gut bacteria and enable estimation of the microbiota-dependency of plasma metabolites. We examined a targeted panel of plasma metabolites, including aromatic amino acid-derived metabolites and TMAO, in individuals with T2D and age- and sex-matched healthy controls before and after 7-day broad-spectrum antibiotic treatment. We aimed to identify microbiota-dependent metabolite differences between the groups and improve understanding of microbial contributions relevant to dietary and microbiota-targeted strategies for T2D prevention and treatment.

## Materials and methods

### Study recruitment

This study consists of post-hoc analyses from the case-control INTENDED (Intestinal imidazole propioNaTE productioN after histiDinE supplementation in healthy and type 2 Diabetes mellitus subjects: role of the gut microbiota) trial. This trial included adults aged 40-70 years with non-insulin dependent T2D (*n* = 20) and age- and sex-matched healthy controls (*n* = 19) of Caucasian or South Asian descent.^36^ Eligible T2D participants had a BMI between 25 and 35 kg/m^2^ and were required to be on stable metformin and statin drug therapy for at least three months prior to inclusion. Healthy controls were required to have a BMI of 19–25 kg/m^2^. Exclusion criteria encompassed use of proton pump inhibitors, use of glucagon-like peptide-1 receptor agonists or insulin, antibiotic exposure within three months prior to enrollment, pregnancy, a prior history of major cardiovascular events and chronic conditions including heart failure, severe renal impairment (eGFR < 30 ml/min), pulmonary, gastrointestinal, hematologic, or other inflammatory diseases. Additional exclusion criteria comprised active infection or malignancy, a history of intestinal surgery, current smoking, a vegetarian diet, excessive alcohol consumption, HbA1c >75 mmol/mol, or concurrent involvement in another clinical trial.

### Study design

All participants received a 7-day broad-spectrum antibiotic regimen, consisting of ciprofloxacin 500 mg once daily, metronidazole 500 mg twice daily, and oral vancomycin 500 mg four times daily. Histidine supplementation (4 g/day, Vital Cell Life L-Histidine 500 mg Capsules, Bunnik, The Netherlands) was initiated two weeks before antibiotic treatment as part of the INTENDED study intervention. As histidine supplementation was administered uniformly across individuals with T2D and healthy controls, it was not included as a covariate in these post-hoc analyses. Data from trial visits performed immediately before and after antibiotic treatment were included in the present study. An overview of the INTENDED trial study design, with these visits highlighted, is provided in Supplementary Figure 1. Fasting blood samples collected at these study visits were used for measurement of targeted plasma metabolite concentrations. Body composition, including total body fat percentage, was assessed at the study visits using bioelectrical impedance analysis (Maltron BF906; Maltron, Rayleigh, UK). Fresh stool samples, obtained within 24 hours before these visits, were used for fecal metagenomic analyses. Dietary intake was recorded for three days prior to each study visit using a digital food diary app (Eetmeter, v4.6.0, Stichting Voedingscentrum Nederland, The Netherlands). Additional details of the study design have been described in the previously published primary manuscript.^36^ The INTENDED trial was conducted in accordance with the principles of the Declaration of Helsinki and received ethical approval from the Academic Medical Center Ethics Committee of the Amsterdam UMC (METC 2019_261). The trial was registered at the Dutch trial registry (registration number NL8372, registration date 11 February 2020). Written informed consent was obtained from all participants prior to enrollment.

### Continuous glucose measurement

In this study, glycemic control was assessed using the FreeStyle Libre 1 sensor and scanner (Abbott, Rungis Cedex, France) for flash glucose monitoring (FGM). FGM data from the two weeks preceding and the two weeks following antibiotic treatment were included. Participants were instructed to perform scans at minimum every 8 hours and to avoid any changes in diet or behavior throughout the monitoring period. Glycemic metrics were derived from the raw FGM data using the CGDA package^37^ in R (v4.5.2).

### Targeted human plasma metabolomics analysis

Targeted metabolomics analysis was performed on plasma samples collected at the study visits before antibiotic treatment (baseline) and after the 7-day antibiotic treatment. The targeted panel included indole-3-propionic acid (IPA), indole-3-acetic acid (IAA), indole-3-lactic acid (ILA), methylindole-3-acetic acid, indoxyl sulfate, serotonin, 5-hydroxyindoleacetic acid, *N*-acetyltryptophan, hippuric acid, 2-hydroxyhippuric acid, 3-hydroxyhippuric acid, 4-hydroxyhippuric acid, 4-hydroxyphenyllactic acid, 4-ethoxyphenyl sulfate, PCS, PAGln, phenylacrylglycine, phenylpropionylglycine, and TMAO. Plasma metabolite levels were measured using stable-isotope-dilution LC-MS/MS as previously described.^18^ ^,^ ^38^ Briefly, 20 μL of plasma was combined with 80 μL ice-cold methanolic solution containing a mixture of internal standards (including D2-indole-3-propionic acid). Samples were vortexed and centrifuged at 21,000 × g for 15 minutes at 4 °C, after which the clear supernatant was collected into glass vials with micro inserts. LC-MS/MS analysis was carried out on a Shimadzu 8050 triple quadrupole mass spectrometer (Shimadzu Scientific Instruments, Columbia, MD, USA). Chromatographic separation was performed on a tandem column setup comprising a Luna Silica column (150 mm × 2.0 mm; 5 μm) (Cat # 00f-4274-B0, Phenomenex, Torrance, CA) and a Kinetex C18 column (50 mm × 2.1 mm; 2.6 μm) (Cat # 00B-4462-AN, Phenomenex, Torrance, CA), eluted with a non-linear gradient using 0.1% propionic acid in water (solvent A) and 0.1% acetic acid in methanol (solvent B). Detection was performed by electrospray ionization in positive mode using multiple reaction monitoring, with transitions m/z 190.00→130.10 for IPA and m/z 191.80→130.10 for D2-IPA. Inter-batch variation, assessed using coefficient of variance, was below 10% across all analytical runs, and data processing was conducted using LabSolutions software (Shimadzu).

### Fecal total genomic DNA isolation and microbiota profiling

For the present study, fecal samples collected at the study visits before and after antibiotic treatment were used. Participants collected fresh stool samples within 24 hours before each study visit. After collection, samples were immediately stored at −20 °C at home and transported to the research facility in a cooling bag, where they were stored at −80 °C until further analysis. Total genomic DNA was extracted from fecal samples using Stool Transport and Recovery (STAR) buffer (Roche, Basel, Switzerland) and repeated bead beating^39^ at 5.5 m s^−1^ with three bursts of 60 seconds each and 20-second intervals between bursts, followed by heating at 95 °C for 15 minutes at 1000 rpm and centrifugation at 14,000 rpm for 5 minutes at 4 °C. A total of 250 µL of the resulting supernatant was transferred for automated DNA purification using the Maxwell RSC Blood DNA Kit (Promega, Leiden, the Netherlands), and DNA was eluted in approximately 60 µL of nuclease-free water.

Sequencing libraries were generated by a PCR-free approach and submitted to Novogene (Cambridge, UK) for sequencing on an Illumina HiSeq platform, yielding 150-bp paired-end reads with a minimum of 6 G data per sample. Full details of the shotgun metagenomic sequencing and data processing have been previously reported.^40^ Briefly, the MEDUSA pipeline was used for metagenomic processing.^41^ Raw reads were processed using the NGless v.10 pipeline, applying quality filtering (Phred score <25 and reads <45 bp removed), and removal of non-microbial contaminants. Filtered reads were subsequently mapped against the Integrated Gene Catalog, containing 9.9 million human gut microbial genes,^42^ using Burrows-Wheeler Alignment.^43^ Gene abundances were computed using the NGless dist1 option, and processed in MetaOminer V1.2 for rarefaction to 10^7^ reads and RPKM normalization. Metagenomic Species (MGS) were defined as Co-Abundance Gene Groups (CAGs) containing more than 500 genes, with relative abundance calculated as the average of the 50 most highly correlating genes. Species-level classification was assigned when at least 50% of MGS genes matched the same NCBI reference genome at 90% length coverage and 95% sequence identity, while genus and phylum levels required 85% and 75% identity, respectively. Taxonomic agglomeration was performed using the phyloseq package (v1.54.0) in R.

### Statistical analysis

Normality was assessed using the Shapiro-Wilk test. Continuous variables are presented as mean ± SD or median [IQR] for normally and non-normally distributed data, respectively. Linear mixed-effects models were used to assess between-group differences in plasma metabolite levels both before and after antibiotic treatment, as well as differences in changes over time, accounting for repeated measurements within participants. *P*-values from all metabolite-level comparisons were adjusted for multiple testing using the Benjamini–Hochberg (BH) procedure to control the false discovery rate (FDR). Group-level trajectories were visualized as median [IQR] across visits. IPA levels were compared between groups using an unpaired t-test. The association between IPA and T2D status was assessed using logistic regression adjusted for age, sex, and total body fat percentage after log transformation of IPA. For clinical correlation analyses, FDR correction was applied across all tested variables.

Alpha and beta diversity were assessed using the Shannon index and Bray–Curtis dissimilarity, respectively. Differences in alpha and beta diversity between the lowest and highest plasma IPA tertiles prior to antibiotic treatment were evaluated using the Wilcoxon rank-sum test and PERMANOVA (1000 permutations), using the vegan package (v2.7-2). As a sensitivity analysis, the proportion of unclassified reads was included as a covariate in the PERMANOVA model and in a linear model of Shannon diversity, to assess whether baseline differences in classification completeness accounted for the observed associations. Longitudinal microbiota analyses were restricted to participants with paired fecal samples available before and after antibiotic treatment (*n* = 22, including 14 participants with T2D and 8 controls). Within-group changes in Shannon diversity before and after antibiotic treatment were assessed using paired Wilcoxon signed-rank tests. Differences in the change in Shannon diversity between healthy controls and participants with T2D were assessed using a Wilcoxon rank-sum test.

Species with a prevalence below 20% were excluded prior to downstream analyses, after which relative abundances were renormalized to 100% within each sample. Species-level relative abundances were CLR-transformed (with a pseudocount of 0.5 × the minimum non-zero abundance) and analyzed using Weighted Gene Co-expression Network Analysis (WGCNA) to identify co-abundance modules. Module eigengenes were subsequently correlated with plasma IPA levels to identify IPA-associated modules, with FDR correction applied across module–trait correlations to define significant modules. Within significant modules, species were considered IPA-associated if they met thresholds of module membership (kME) > 0.5 and gene significance (GS) > 0.4. To complement this network-based approach, relative abundances were used as input for a Random Forest regression model (1000 trees) to predict plasma IPA levels. Given the small sample size (*n* = 39), model performance was evaluated using out-of-bag estimates and leave-one-out cross-validation (LOOCV) to obtain less biased performance estimates. To ensure stable importance rankings, the model was run across five random seeds, and species were ranked by mean permutation importance. Species with a coefficient of variation ≥ 0.5 across seeds were excluded, and the top 15 remaining species were selected based on this stability-filtered mean ranking. Results from both approaches were compared.

Spearman rank correlations were used to test associations between HUMAnN3 pathway abundances and plasma IPA levels. Pathways present in ≥ 25% of samples were included. ANCOM-BC2 was additionally applied to adjust for age and sex, with IPA included as a continuous covariate and multiple testing correction using the BH procedure (q < 0.05). Pseudo-count sensitivity analysis was performed to assess whether associations were robust to zero-count handling.

Statistical significance was defined as *p* < 0.05, or FDR-corrected *p* < 0.05 where multiple testing correction was applied. All statistical analyses were performed in R (v4.5.2).

## Results

### Participants baseline characteristics

This study included 20 participants with T2D and 19 age- and sex-matched healthy controls. Baseline characteristics of all participants are presented in Table 1. BMI was significantly higher in the T2D group compared with healthy controls, consistent with the study inclusion criteria. Total cholesterol, LDL, and HDL levels were higher in the control group, which may reflect the use of statins among the participants with T2D, in contrast to the medication-naïve control group.

**Table 1.** Baseline characteristics.

Characteristics*	Type 2 Diabetes (*n* = 20)	Control (*n* = 19)	*p*-value
**Demographic/Anthropometric**			
Age (years)	61 [54–65]	63 [54–68]	0.44
Female	9 (45.0)	10 (52.6)	0.88
BMI (kg/m^2^)	29.28 ± 2.94	25.74 ± 2.22	<0.001
**Laboratory parameters**			
AST (U/L)	21 [19–24]	21 [20–29]	0.91
ALT (U/L)	21 [15–27]	21 [17–25]	0.89
GGT (U/L)	23 [19–27]	14 [12–21]	0.001
HbA1c (mmol/mol)	54 [50–61]	35 [34–37]	<0.001
Fasting glucose (mmol/L)	8.2 [7.4–9.3]	5.3 [5.1–5.6]	<0.001
Fasting Insulin (pmol/L)	56 [41–100]	38 [28–55]	0.020
Triglycerides (mmol/L)	1.05 [0.86–1.38]	0.78 [0.59–1.23]	0.092
Total cholesterol (mmol/L)	3.53 [2.82–4.57]	5.65 [4.54–5.97]	<0.001
HDL-cholesterol (mmol/L)	1.18 [1.07–1.58]	1.55 [1.26–1.62]	0.023
LDL-cholesterol (mmol/L)	1.67 [1.33–2.46]	3.57 [2.72–3.95]	<0.001

^*^ Mean ± SD or median [IQR] for continuous variables and *n* (%) for categorical variables.

### Gut microbiota-derived plasma indole-3-propionic acid is negatively associated with T2D in humans

Building on previous research linking gut-dependent pathways to cardiometabolic disease,^14^ ^,^ ^18^ we quantified targeted aromatic amino acid-derived metabolites and TMAO before and after broad-spectrum antibiotic exposure. This approach enabled us to compare pre-antiobiotic profiles between participants with T2D and healthy controls while directly establishing the extent of gut microbial contribution. Antibiotic-mediated depletion of the gut microbiota was highly effective in both T2D participants and healthy controls, as demonstrated by the significant reduction in circulating gut-derived metabolite levels, including TMAO, PCS, PAGln, phenylacryl glycine, indoxyl sulfate, IPA, hippuric acid, 3-hydroxyhippuric acid and 4-hydroxyhippuric acid (Supplementary Figure 2). Consistently, Shannon diversity decreased significantly with antibiotic treatment in both healthy controls (*p* = 0.008) and participants with T2D (*p* < 0.001), and this decrease did not differ between groups (*p* = 0.97), confirming comparable microbiota depletion (Supplementary Figure 3). Among all metabolites measured in the pre-antibiotic samples, only IPA exhibited a statistically significant difference between groups (Supplementary Figure 2). Therefore, subsequent analyzss focused on IPA to investigate the effects of antibiotic treatment and to further characterize its clinical and microbial associations. Pre-antibiotic IPA levels were markedly higher in healthy controls (1.56 [1.08-2.02] µM) than in participants with T2D (0.56 [0.47-0.85] µM) (Figure 1a). Following oral antibiotic treatment, IPA was reduced to near-undetectable levels in both groups (Figure 1b), confirming its predominantly gut microbiota-derived origin. Furthermore, higher IPA levels were independently associated with reduced odds of T2D after adjustment for age, sex and total body fat percentage (OR per log-unit increase in IPA: 0.20, 95% CI: 0.05–0.81) (Figure 1c), highlighting IPA as a microbial metabolite associated with lower T2D risk.

**Figure 1.**
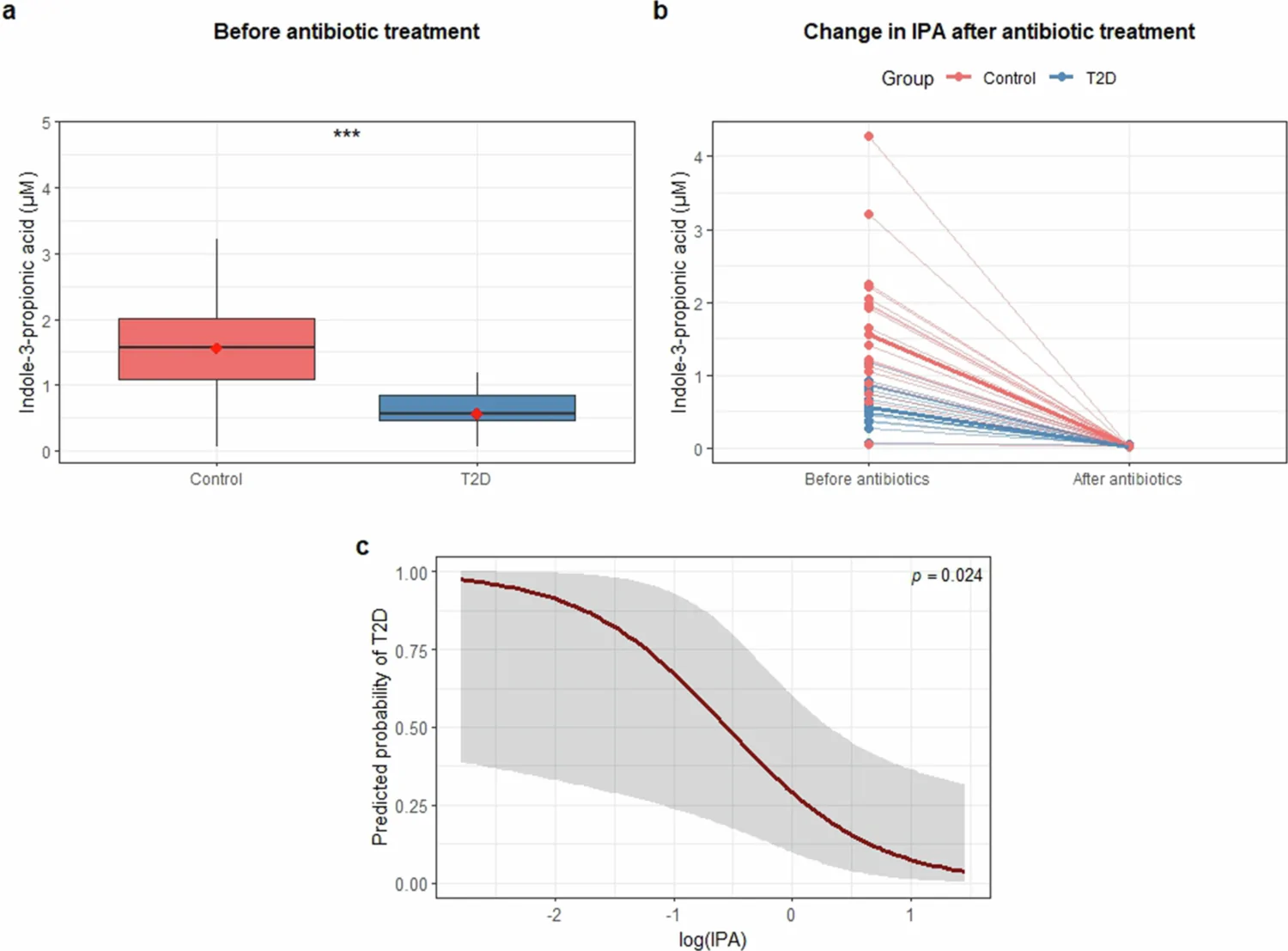
Plasma indole-3-propionic acid is negatively associated with T2D and virtually exclusively gut microbiota-derived in humans. (a) Fasting plasma IPA concentrations before antibiotic treatment in participants with T2D (*n* = 20) and healthy controls (*n* = 19) ***p < 0.001. (b) Fasting plasma IPA concentrations before and after one week of antibiotic treatment. Lines connect measurements within each participant (*n* = 39). IPA levels are reduced to near-undetectable levels in all participants, regardless of group. (c) Logistic regression (adjusted for age, sex and total body fat percentage; *n* = 38) shows that each unit increase in log-transformed IPA is associated with reduced odds of T2D (OR: 0.20, 95% CI: 0.05–0.80). IPA, indole-3-propionic acid; T2D, type 2 diabetes.

### Plasma IPA inversely correlates with markers of metabolic dysfunction including glycemic variability

Plasma IPA levels were inversely correlated with cardiometabolic risk markers, showing strong associations with HbA1c (*ρ* = −0.64, *p* < 0.001; Figure 2a) and FGM-derived mean glucose (*ρ* = −0.62, *p* = 0.0011; Figure 2b). Moderate inverse correlations were observed with fasting glucose (*ρ* = −0.58, *p* < 0.001; Figure 2c), BMI (*ρ* = −0.57, *p* < 0.001; Figure 2 d), gamma-glutamyl transferase (GGT) (*ρ* = −0.45, *p* = 0.0042; Figure 2e), and leukocyte count (*ρ* = −0.44, *p* = 0.0048; Figure 2f). IPA displayed weaker, though still statistically significant, inverse correlations with C-peptide (*ρ* = −0.39, *p* = 0.0157; Figure 2g) and glycemic variability (MAGE; *ρ* = -0.38, *p* = 0.0421; Figure 2h).

**Figure 2.**
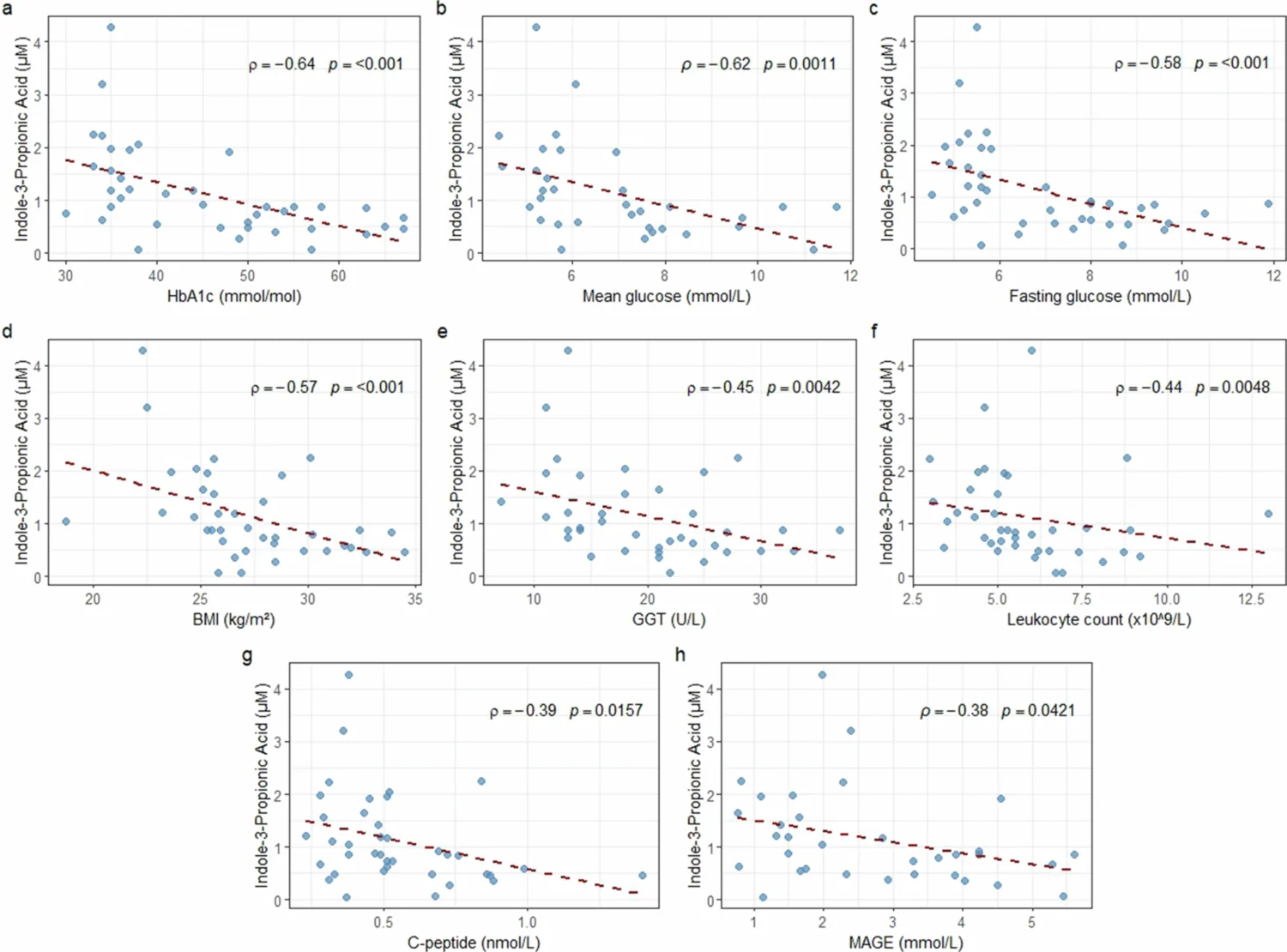
Plasma IPA is inversely correlated with markers of metabolic dysfunction including glycemic variability. (a−h) Spearman correlations between fasting plasma indole-3-propionic acid (IPA) concentrations and clinical parameters before antibiotic treatment. Correlation coefficients (*ρ*) and FDR-adjusted *p*-values are shown within each panel, including HbA1c (a; *n* = 39), mean glucose assessed by flash glucose monitoring (b; *n* = 33), fasting glucose (**c**; *n* = 39), BMI (d; *n* = 38), GGT (e; *n* = 39), leukocyte count (f; *n* = 39), C-peptide (g; *n* = 39), and MAGE assessed by flash glucose monitoring (h; *n* = 33). Abbreviations: BMI, body mass index; MAGE, mean amplitude of glycemic excursions.

### Fasting plasma IPA levels are associated with differential gut microbiota composition and species co-abundance patterns

Given the gut microbial origin of plasma IPA, we next examined pre-antibiotic associations between plasma IPA levels and fecal gut microbiota. For diversity analysis, participants were stratified into tertiles based on fasting plasma IPA levels, comparing individuals in the highest and lowest tertiles. Alpha diversity did not differ between IPA tertiles (Figure 3a). In contrast, beta diversity differed significantly between the top and bottom tertiles (R^2^ = 0.069, *p* = 0.006) (Figure 3b), indicating an association between plasma IPA and gut microbial community structure. This association remained significant in a sensitivity analysis adjusting for the proportion of unclassified reads (R^2^ = 0.059, *p* = 0.029), indicating that classification completeness did not confound the result.

**Figure 3.**
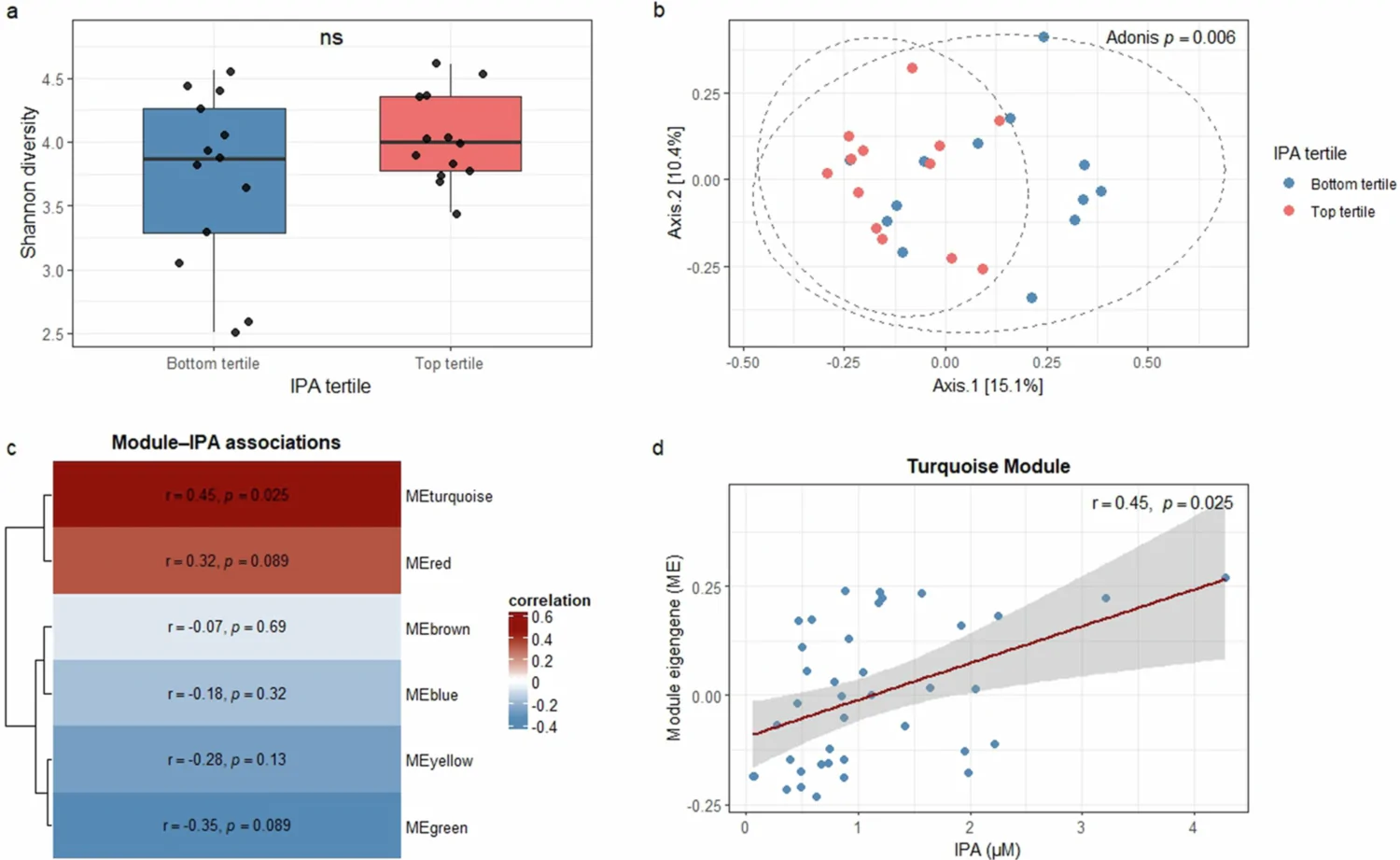
Fasting plasma IPA levels are associated with differential gut microbiota composition and species co-abundance patterns. (a) Alpha diversity, assessed by Shannon diversity, in participants from the bottom (*n* = 13) and top (*n* = 13) tertiles of fasting plasma IPA levels before antibiotic treatment. Statistical significance was assessed using the Wilcoxon rank-sum test. (b) Beta diversity based on Bray–Curtis dissimilarity at species level, visualized by principal coordinates analysis (PCoA). Group differences between the bottom and top IPA tertiles were assessed using PERMANOVA. (c) Heatmap of Pearson correlations between module eigengenes and continuous fasting plasma IPA levels (*n* = 39) prior to antibiotics. Correlation coefficients (r) and FDR-adjusted *p*-values are shown within each cell, highlighting the turquoise module as the only module demonstrating a significant correlation with plasma IPA. (d) Association between the turquoise module eigengene and plasma IPA levels. Abbreviations: ns, not significant; IPA, indole-3-propionic acid.

To further explore the microbial features associated with plasma IPA variation, we applied weighted gene co-expression network analysis (WGCNA) to identify co-abundance modules of microbial species and assess their associations with plasma IPA levels. One module (the turquoise module) showed significant positive correlations with plasma IPA (Figure 3c−d), suggesting that specific compositions of gut microbial species are associated with the interindividual variation in plasma IPA.

### Identification of IPA-associated microbial species through network analysis and Random Forest modeling

To identify specific species contributing to this module-IPA correlation, hub species within the turquoise module were defined using thresholds for module membership (kME > 0.5) and individual correlation with IPA (gene significance (GS) > 0.4), identifying 24 species in this module (Figure 4a). No species in the other co-abundance modules showed a GS > 0.4, indicating that the strongest IPA-associated species were concentrated within the turquoise module. Taxonomic classification of the 24 turquoise module hub species showed that Lachnospiraceae, Oscillospiraceae, and Clostridiaceae were the most represented families (Figure 4b). A Random Forest model was applied to species-level microbiota profiles to complement findings from WGCNA and assess the relative importance of individual microbial species for plasma IPA concentrations. The top 15 species ranked by permutation importance are shown in Figure 4c. Eight of these top-ranked species were also identified as turquoise module hub taxa (Figure 4a), highlighting these as consistent candidate taxa linked to IPA across both analytical approaches: two Oscillospiraceae (*GGB9737_SGB15309* and *GGB9616_SGB15052*), one Clostridiaceae (*Clostridium* sp. AF20-17LB), one unclassified Clostridia (*GGB13404_SGB14252*), one Lachnospiraceae (*Coprococcus eutactus*), one unclassified Eubacteriales (*GGB3351_SGB4434*), and two species of unspecified family affiliation (*GGB51441_SGB71759* and *GGB49418_SGB69331*).

**Figure 4.**
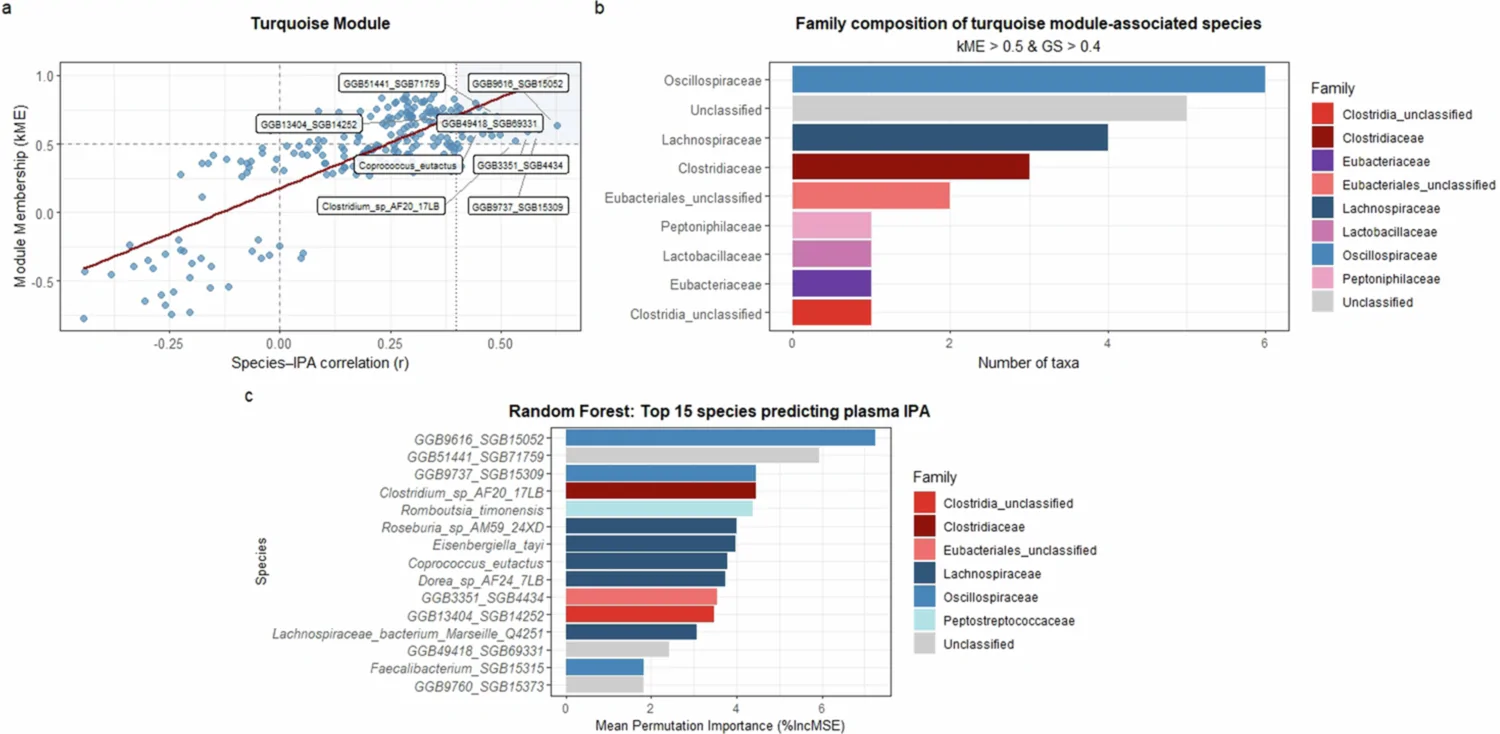
Identification of IPA-associated microbial species through network analysis and Random Forest modeling. (a) Scatter plot of gene significance (GS; Pearson correlation with plasma IPA) against module membership (kME) for species in the turquoise co-abundance module. The highlighted region indicates hub species meeting both thresholds (GS > 0.4, kME > 0.5). Species identified in both the turquoise module and the top 15 Random Forest features (c) are labeled. (b) Family-level taxonomic composition of turquoise module hub species (kME > 0.5, GS > 0.4). (c) Mean permutation importance of the top 15 stable species (CV < 0.5 across five random seeds) from the Random Forest regression model predicting fasting plasma IPA levels. Abbreviations: GS, gene significance (Pearson correlation with plasma IPA); kME, module eigengene connectivity; IPA, indole-3-propionic acid.

In addition to the species-level analyses, we assessed whether functional pathway abundances were associated with plasma IPA. HUMAnN3 pathway abundances were correlated with plasma IPA levels using Spearman rank correlations and further evaluated using ANCOM-BC2 adjusted for age and sex, but no significant pathway associations were identified after FDR correction.

## Discussion

Although the gut microbial origin of IPA has been established in preclinical models and healthy humans,^18^ ^,^ ^26^ ^,^ ^44^ ^,^ ^45^ this is the first intervention study in individuals with T2D demonstrating a reduction of plasma IPA to near-undetectable levels following broad-spectrum antibiotic–induced microbiota suppression, providing causal evidence of its predominantly microbial origin in this disease context. Pre-antibiotic IPA concentrations were comparable to previously reported levels (1.0 μM) in healthy adults,^46^ supporting the biological relevance of the observed range and its reduction after microbiota suppression.

Higher plasma IPA levels have previously been associated with lower insulin resistance, fasting glucose,^47^ and reduced T2D risk,^48^ with experimental studies further suggesting a role in improving glucose homeostasis.^49^ ^,^ ^50^ Consistent with these findings, we observed lower fasting plasma IPA levels in participants with T2D compared with healthy controls, and higher IPA concentrations were associated with reduced odds of T2D. Furthermore, plasma IPA was inversely correlated with HbA1c and fasting glucose, and we provide novel evidence extending these inverse associations to FGM-derived measures of glycemic variability. Given that glycemic variability is associated with insulin resistance and may serve as an early marker of T2D risk,^51^ this finding supports a possible protective association between IPA and T2D. Beyond glycemic measures, IPA was inversely correlated with BMI, GGT, and leukocyte count, consistent with prior evidence linking low plasma IPA to obesity,^49^ ^,^ ^52^ low-grade inflammation,^49^ and experimental data showing that IPA supplementation reduces plasma lipids and attenuates steatohepatitis.^49^ ^,^ ^50^ ^,^ ^53^ ^,^ ^54^ Proposed mechanisms include anti-inflammatory immune modulation and enhancement of intestinal homeostasis including intestinal barrier integrity through pregnane X receptor (PXR) activation,^55^ aryl hydrocarbon receptor (AhR) signaling,^56^ and modulation of CD4+ T-cell metabolism.^57^

IPA is a microbiota-derived product of tryptophan metabolism^58^ and should therefore be interpreted within the broader host–microbial metabolic network. Other microbial indole derivatives also contribute to intestinal barrier integrity and immune homeostasis, acting in part through AhR and PXR signaling.^59-64^ In addition, dysregulation of host tryptophan metabolism, including increased indoleamine 2,3-dioxygenase (IDO1) activity, alterations in the kynurenine pathway, and changes in serotonin metabolism, has been associated with chronic inflammation, insulin resistance, and T2D.^65-69^ Our targeted panel included several microbial and host-derived tryptophan metabolites beyond IPA. Following antibiotic-mediated microbiota depletion, IPA, IAA, and indoxyl sulfate levels decreased, consistent with disruption of microbial indole metabolism. In contrast, ILA increased following antibiotic treatment. This potentially reflects shifts in microbial community composition, including the increased abundance of the genus *Lactobacillus* observed after treatment,^36^ as certain *Lactobacillus* strains have previously been shown to produce ILA.^70^ ^,^ ^71^ Serotonin and *N*-acetyltryptophan remained unchanged, suggesting limited effects on these host-regulated pathways. As our panel did not include tryptophan and kynurenine, the contribution of host kynurenine pathway activity could not be assessed.

In this study, plasma IPA levels were significantly associated with beta diversity, underscoring a relationship between circulating IPA and gut microbial composition. Supporting this, a previous human cohort study showed that gut microbiota composition explains substantially more interindividual variation in IPA than dietary tryptophan intake itself.^47^ IPA biosynthesis has been linked to the phenyllactate dehydratase (*fldABC*) gene cluster, with *Clostridium sporogenes* representing the best-characterized producer.^26^ ^,^ ^72^ ^,^ ^73^ Additional IPA-producing species, including *Clostridium botulinum,* three strains of *Clostridium cadaveris*, *Peptostreptococcus anaerobius* and *Peptostreptococcus russelli,* ^72-74^ all harbor homologous *fldABC* gene clusters,^72^ ^,^ ^74^ supporting a conserved biosynthetic pathway across phylogenetically distinct anaerobic bacteria. Notably, none of the species identified by our WGCNA or Random Forest analyses are established *fldABC*-carrying IPA producers. Identified IPA-associated species predominantly belonged to the Lachnospiraceae, Oscillospiraceae, and Clostridiaceae families, sharing the anaerobic, fermentative properties of known IPA producers, although our data do not support their direct contribution to IPA biosynthesis. The strongest IPA-associated species clustered within a single co-abundance module, suggesting that circulating IPA may depend on coordinated microbial community structure rather than the IPA-producing capacity of individual species alone. This is consistent with prior evidence that probiotic supplementation with *Bifidobacterium* species increases plasma IPA levels in healthy elderly individuals,^75^ supporting our hypothesized broader role for gut microbial community composition in regulating circulating IPA concentrations.

Eight species were identified across both network analysis and Random Forest modeling. Although none are established IPA producers, we propose several possible indirect roles underlying their association with IPA, based on previous studies. These include modulation of tryptophan availability, and maintenance of intestinal barrier integrity and homeostasis. For example, *C. eutactus* has been implicated in maintaining intestinal homeostasis by promoting gut barrier integrity, reducing intestinal inflammation, and increasing colonic tryptophan availability in murine models.^76-78^ Four of the identified species, including *Clostridium* sp. AF20-17LB, *C. eutactus*, *GGB51441_SGB71759* and *GGB9616_SGB15052*, have previously been reported to be more abundant in individuals consuming plant-based diets.^79^ Such diets are typically enriched in dietary fiber and polyphenols,^80^ ^,^ ^81^ both of which have been independently associated with higher plasma IPA concentrations.^82-84^ Collectively, these findings suggest that dietary fiber and polyphenols, commonly enriched in plant-based diets, may promote IPA production primarily through their effects on gut microbiota composition.

It is important to note that all participants received histidine supplementation as part of the INTENDED trial. Histidine and tryptophan share overlapping intestinal transporters,^85^ raising the possibility of competitive uptake affecting luminal tryptophan availability to IPA-producing taxa. Histidine supplementation was previously shown to alter gut microbial composition in this trial, with several genera changing in abundance and correlating with histidine levels.^36^ None, however, are recognized IPA-producing genera. As supplementation was consistent across pre- and post-antibiotic visits, it is unlikely to account for within-person IPA reduction after microbiota suppression, but effects on pre-antibiotic levels and the reported associations cannot be excluded. Metformin use, part of the inclusion criteria for T2D participants in this trial, represents another potential confounder. Metformin is recognized as a modulator of gut microbiota composition and microbial metabolism,^86-88^ and may therefore have contributed to differences observed between T2D and control groups. However, metformin has been reported to partially restore serum IPA levels in a T2D mouse model,^89^ suggesting that it would be expected to attenuate rather than explain the lower IPA levels observed in our T2D participants. Nevertheless, an independent effect of metformin on IPA-associated microbial taxa or other metabolic parameters cannot be ruled out.

A strength of this study is the use of broad-spectrum antibiotic treatment to suppress the gut microbiota in both healthy controls and individuals with T2D, enabling assessment of microbiota-dependent metabolite changes across these populations. The inclusion of age- and sex-matched healthy controls strengthens the comparability between groups. Furthermore, the combination of targeted plasma metabolomics, metagenomic analyses, and clinical data enabled comprehensive characterization of the relationship between gut microbiota composition, plasma IPA levels, and clinical characteristics in humans.

However, several limitations should be considered. The relatively small sample size may limit statistical power and generalizability, particularly for the microbiota analyses, where the high number of microbial features relative to sample size can make Random Forest and WGCNA results unstable. Despite repeated modeling, LOOCV, and stability filtering across seeds, these results should be considered exploratory and hypothesis-generating, and need validation in larger cohorts. The limited sample size likely also explains why functional pathway analysis showed no significant associations with plasma IPA, which may reflect insufficient power rather than a true absence of biological signal. Antibiotic treatment frequently induced diarrhea, which may have transiently altered gut transit time, nutritional status, intestinal permeability, host metabolism, and microbial metabolite absorption, potentially affecting circulating metabolite levels independently of microbial depletion. These effects, however, do not affect the core finding that IPA is predominantly microbially derived, nor the clinical and microbiome associations with IPA reported here, as both were assessed using pre-antibiotic data. Overall caloric intake, assessed by food diaries, remained stable throughout the study. Yet, intake of specific IPA-related nutrients such as fiber, polyphenols, and protein was not separately assessed and cannot be excluded as a contributing factor. Additionally, our targeted metabolomics panel did not include tryptophan or kynurenine, precluding assessment of potential alterations in host tryptophan metabolism, including the kynurenine pathway.

In conclusion, this study identifies IPA as metabolite reduced in individuals with T2D compared with healthy controls, and provides direct human evidence of its virtually exclusive gut microbial origin, extending previous findings in healthy individuals to a clinically relevant context. Higher IPA levels were associated with improved cardiometabolic measures, including novel FGM-derived metrics of glycemic variability, supporting a potential protective association between IPA and T2D. The identified microbial associations suggest that circulating IPA may reflect broader gut microbial community characteristics rather than the IPA-synthesizing potential of individual taxa alone. Building on prior literature, we hypothesize that IPA-associated species contribute indirectly to plasma IPA levels, for example through modulation of tryptophan availability or maintenance of gut barrier integrity and intestinal homeostasis. Given the modest cohort size, these microbiota findings require validation in larger, independent cohorts. Collectively, this study underscores the relevance of gut microbiota-derived IPA in metabolic health and T2D and provides a direction for future intervention studies exploring whether dietary or microbiome-targeted strategies can modulate IPA and ultimately contribute to T2D prevention and treatment.

## Supplementary Material

Supplementary Material Supplementary Material. Figures docx

Supplementary Figure 2.jpeg Supplementary Figure 2.jpeg.

Supplementary Material Supplementary Figure 1.jpeg.

Supplementary Material Supplementary Figure 3.jpeg

## Data Availability

Access to some of the data generated or analyzed in this study is restricted to protect patient confidentiality or because the data are subject to licensing agreements. Upon reasonable request, the corresponding author will provide details of these restrictions and explain the conditions under which access to the data may be granted.
