# Supplementary material for: Plasma indole-3-propionic acid is a gut-derived metabolite and is associated with type 2 diabetes and cardiometabolic outcomes: Evidence from a human antibiotic intervention
Source: Gut Microbes. 2026 Sep 26;18(1):2736321. doi: 10.1080/19490976.2026.2736321 (PMC13618833; doi:10.1080/19490976.2026.2736321)
Supplement: Supplementary file 1 [file KGMI_A_2736321_SM9472.docx]

**Supplementary Figure 1. INTENDED trial study design, with study visits included in the present post-hoc analysis highlighted.** T2D, type 2 diabetes; AB, antibiotics.

**Supplementary Figure 2. Plasma concentrations of targeted plasma metabolites before and after antibiotic treatment.**
Median plasma concentrations and interquartile ranges of the targeted plasma metabolites are shown for participants with T2D and healthy controls, before and after antibiotic treatment. Lines connect group medians across visits. Between-group differences at each timepoint were assessed using linear mixed-effects models, accounting for repeated measures within participants. *P*-values were adjusted for multiple testing using the Benjamini-Hochberg procedure. **, p < 0.01; ***, p < 0.001. T2D, type 2 diabetes.

**Supplementary Figure 3. Antibiotic treatment reduces Shannon alpha diversity to a comparable extent in healthy controls and participants with T2D.**
Boxplots show Shannon alpha diversity before and after antibiotic treatment. Diversity declined significantly in both healthy controls (*p* = 0.008) and participants with T2D (*p* < 0.001), with no significant difference in the extent of reduction between groups (*p* = 0.97), indicating similar depletion of the gut microbiota across both populations.
**, p < 0.01; ***, p < 0.001. T2D, type 2 diabetes.
